# Microstructure and Recrystallization Behavior of Heating Rate-Controlled Electrolytic Capacitor Aluminum Foil under Cold Forming and Annealing

**DOI:** 10.3390/ma16114128

**Published:** 2023-06-01

**Authors:** Yunlei Wang, Taibin Wu, Luchang Che, Guangjie Huang

**Affiliations:** 1 College of Materials Science and Engineering, Chongqing University of Arts and Sciences, Chongqing 402160, China; 2 Department of Advanced Materials and Technology, China Ordnance Industry 59th Research Institute, Chongqing 400039, China; 3 College of Materials Science and Engineering, Chongqing University, Chongqing 400044, China

**Keywords:** electrolytic capacitor, aluminum foil, microstructure, recrystallization

## Abstract

A novel annealing process of controlled heating rate is used to produce severe cold-formed aluminum plates, which are processed into aluminum foil and mainly used for high-voltage electrolytic capacitor anodes. The experiment in this study focused on various aspects such as microstructure, recrystallization behavior, grain size, and grain boundary characteristics. The results revealed a comprehensive influence of cold-rolled reduction rate, annealing temperature, and heating rate on recrystallization behavior and grain boundary characteristics during the annealing process. The heating rate applied plays a crucial role in controlling the recrystallization process and the subsequent grain growth, which ultimately determines whether or not the grains will become larger. In addition, as the annealing temperature rises, the recrystallized fraction increases and the grains size decreases; conversely, the recrystallized fraction decreases as the heating rate increases. When the annealing temperature remains constant, the recrystallization fraction increases with a greater deformation degree. Once complete recrystallization occurs, the grain will undergo secondary growth and may even subsequently become coarser. If the deformation degree and annealing temperature remain constant, the increased heating rate will result in a lower recrystallization fraction. This is due to the inhibition of recrystallization, and most of the aluminum sheet even remains in a deformed state before recrystallization. This kind of microstructure evolution, grain characteristic revelation, and recrystallization behavior regulation can provide effective help for enterprise engineers and technicians to guide the production of capacitor aluminum foil to a certain extent, so as to improve the quality of aluminum foil and increase the electric storage performance.

## 1. Introduction

The electrolytic capacitor is a fundamental electronic component that holds significant importance in various electronic settings and electrical circuits [1,2]. Its functions include blocking, low-frequency filtering, energy storage, and coupling [3,4,5]. Additionally, it plays a crucial role in specific circuits, such as frequency division circuits, pulse circuits, sequence circuits, and energy storage circuits, and aluminum foil is mainly used to manufacture electrolytic capacitors [3,4,6,7,8]. As a result, it finds extensive applications in communication equipment, digital products, machinery, automotive electronics, household appliances, and industrial frequency conversion [9,10]. With the rapid growth of the global electronics industry and the adjustment of the international electronics industry in the twentieth century, the production of electronic products has increased significantly, and the pace of product upgrading has accelerated.

At present, the global aluminum electrolytic capacitor supply market is becoming more and more mature, and is mainly concentrated in Japan, mainland China, Taiwan, and South Korea. The Asia Pacific region is expected to hold a significant portion of 58% of the worldwide market. The market for aluminum electrolytic capacitors was valued at CNY 43.1 billion in 2019 and is projected to increase to CNY 51.7 billion by 2026, with a compound annual growth rate (CAGR) of 2.6% [11,12,13,14,15]. However, China’s aluminum electrolytic capacitor manufacturing enterprise market mainly produces consumer products such as flat-screen TVs, monitors, computers, and ordinary industrial products [16]. Electrolytic capacitors are a type of capacitor that utilizes an oxide film formed on the metal surface through electrochemical means as a dielectric. Common electrolytic capacitors in the market include ceramic electrolytic capacitors, staggered electrolytic capacitors, silver electrolytic capacitors, and thin film electrolytic capacitors [3,4,17,18]. Among them, aluminum electrolytic capacitors are widely used in the field of electronic circuits, given their benefits of large voltage range, small size, large storage capacity, and high performance [19].

Summarizing previous studies, the purpose of Liu et al. [20]’s research is to investigate how branch pits and tunnels form and increase the specific surface area and capacitance of anode aluminum foil for high-voltage electrolytic capacitors through D.C. etching in both acidic and neutral solutions. The capacitance and equivalent series resistance of these components have significant effects on the performance and reliability of power electronic systems. Using additive manufacturing technology, anode foils were created for aluminum electrolytic capacitors. The impact of particle size and sintering temperature on the anode foil was studied [21], and the findings revealed that the sintering neck and particle size were crucial in determining the electrical characteristics of the produced powder foil. Pan et al. [22] examined the process of creating anodic aluminum foil, which involves several steps such as hydration, formation, heat treatment, and phosphoric acid treatment. This study focuses on the changes in the microstructure of the oxide film during the preparation process, and the findings indicate that the area of pores on the surface of the foil decreases progressively after hydration and formation. Other studies focus on topics such as the preparation of electrolytes, capacitor material selection, and corrosion problems [23,24,25].

After analyzing the background of capacitor aluminum foil, and based on the research status, the primary focus of this paper is to examine how the grain size, microstructure, and recrystallization behavior of aluminum foil are impacted by cold forming, temperature, and heating rate. By conducting this research, we can identify the trends in the development of microstructure and the way it recrystallizes, and aim to create fundamental models that link microstructure and property compatibility. Finally, an organizational performance relationship model is established.

## 2. Materials and Methods

### 2.1. Materials

**Figure 1:**
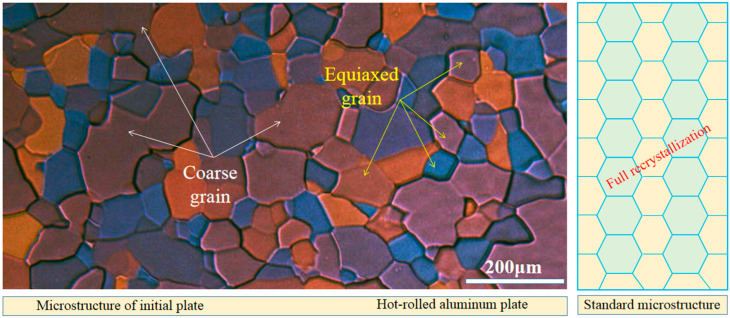
Microstructure of the initial sheet in hot-rolled state.

The chemical composition of the aluminum plate received for analysis consisted primarily of 99.99% aluminum (Al), with trace amounts of iron (Fe), copper (Cu), magnesium (Mg), silicon (Si), manganese (Mn), nickel (Ni), zinc (Zn), and titanium (Ti). A detailed breakdown of the chemical composition is provided. The initial aluminum plates used in this study were sourced from Southwest Aluminum (Group) Co., Ltd. (Chongqing, China), and were initially three-layered liquid electrolysis, hot-rolled, high-purity aluminum plates, as shown in Figure 1. The chemical composition was analyzed using a PDA-6000 photoelectric spectrometer (Shimadzu, Chengdu, China). The impurity elements present in the aluminum matrix were in the form of a solid solution, and their effect on recrystallization and grain growth was deemed negligible.

The completion of the recrystallization of the original aluminum plate is evident in Figure 1, where the grains are now equiaxed, although some of them have grown abnormally, and the average grain size is ~125 μm. The aluminum alloy in this condition serves as the fundamental material for further investigation. Some processes involved in this include cold rolling (CR), intermediate annealing, secondary cold rolling, and finished annealing. Based on these, it is easy to analyze the microstructure and future properties of a cold-rolled aluminum sheet, with the aim of applying these to improve engineering practice.

### 2.2. Methods

**Figure 2:**
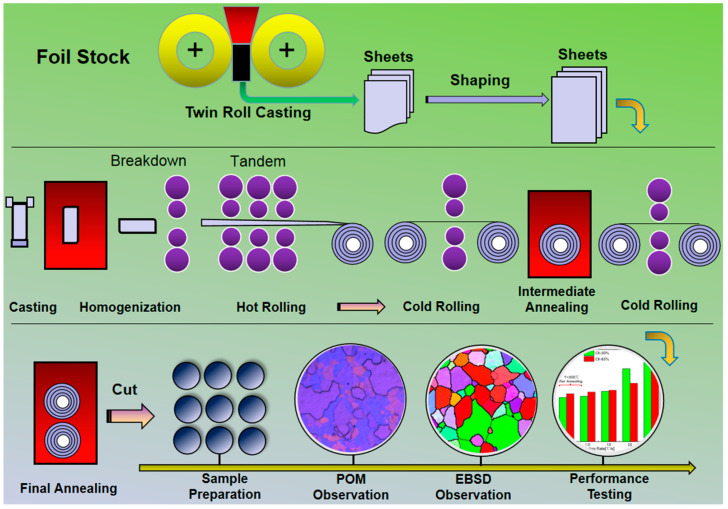
Process schedule for foil stock production and microstructure characterization.

Once the chosen aluminum alloy plate undergoes pre-treatment, it will be subjected to processes such as shaping, trimming, and deburring. The specific preparation flowchart is shown in Figure 2. This indicates the scheduling of processes for the production of foil stock and the analysis of its microstructure.

Figure 2 illustrates the process steps involved in this research, which include cold rolling and heat treatment. A high-purity aluminum plate with a thickness of 7.6 mm was initially hot-rolled and then cold-rolled to varying degrees of deformation, namely 50%, 70%, and 85%. Each cold-deformed plate was then heated to a target temperature of 350–500 °C at different heating rates to complete the annealing process. This study focused on the recrystallization volume fraction, crystallite dimension, and grain boundary characteristics, which were adjusted by varying the heating rate.

The selected samples that had been annealed and treated with anodic coating and electropolishing and their microstructure were examined using a polarizing optical microscope (POM) (AOSVI, Shenzhen, China). The experimental samples were first cut into 10 mm × 5 mm × 0.11 mm pieces and the cross-sectional surfaces of RD/ND or RD/TD were mechanical polished and observed with the POM, equipped with a WF10X large-field high-point flat-field eyepiece, field of view φ 22 mm, 45° oblique cutting head. At the time of the experiment, we ensured that the laboratory was shockproof, moisture-proof and dust-proof, the power supply was 220 V, 50 HZ, and the temperature was 0 to −40 °C.

Micro-grain orientation images were obtained through electron back-scattered diffraction (EBSD) with a Zeiss (Oberkochen, Germany) Supra 55 scanning electron microscope (SEM). Specimens of the detected areas were 300 µm × 500 µm, and the step size was set to 0.5 µm, the EBSD mapping was prepared through mechanical polishing down to colloidal Al_2_O_3_, and samples for BSE observation were slightly etched using Keller reagent (consist of 2 mL HF, 3 mL HCl, 5 mL HNO_3_, and 190 mL H_2_O) after mechanical polishing. The electropolishing solution was made up of a mixture of perchloric acid and alcohol, with liquid nitrogen added to cool it down. The ratio of HClO_4_ to C_2_H_5_OH was 1:9. During electrolytic polishing, the positive electrode of the power supply was connected to the sample, while the negative electrode was connected to stainless steel. The standard parameters were 18 V for voltage, 0.10–0.25 A for current, and 30–45 s for time. The anodic coated liquid consisted of 5 mL HBF4 and 200 mL H_2_O, with a voltage of 12 V, current of 0.01–0.10 A, and time of 15–30 s. According to the actual situation of this experiment, the principle of multiple laminating with a small current was used to achieve a better laminating effect, which clearly showed the grain boundary characteristics.

## 3. Results and Analysis

### 3.1. Microstructure Observation

Among the uses of aluminum foil, we know that high-purity aluminum foil is the key material for manufacturing electrolytic capacitors. The microstructure and cubic texture content of this material are the primary factors that determine the specific capacitance of the capacitors. The annealing process is a critical step in the production of high-purity aluminum foil, as it significantly impacts the material’s microstructure and texture. Despite the fixed chemical composition and rolling deformation process, annealing plays a crucial role in the recrystallization and kinetics of the finished foil.

**Figure 3:**
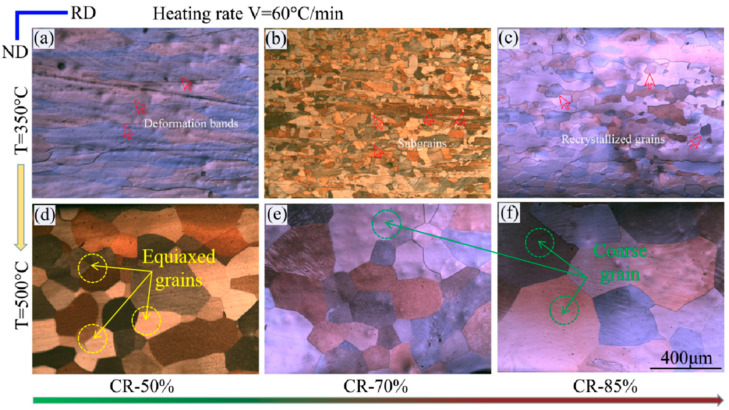
POM image of microstructure under (**a**–**c**) T = 350 °C, V = 60 °C/min, and CR-50%, CR-70%, CR-85%, respectively; (**d**–**f**) T = 500 °C, V = 60 °C/min, and CR-50%, CR-70%, CR-85%, respectively.

In order to achieve our research objectives, we conducted an examination of the microstructure under varying deformation degrees, annealing temperatures, and heating rates. Through this analysis, we gained insight into the evolution law of the microstructure and texture, as well as the impact of deformation degrees, annealing temperatures, and heating rates on the recrystallization behavior. Figure 3 shows POM images of microstructure under CR-50%, CR-70%, and CR-85%, respectively. In effect, this is a form of birefringence, showing different colored grains in order to distinguish between differently oriented grains. In Figure 3a–c, T = 350 °C and V = 60 °C/min, while in Figure 3d–f, T = 500 °C and V = 60 °C/min. When the annealing temperature is 350 °C and V is 60 °C/min, as the degree of deformation increases, the percentage of recrystallized grains of the aluminum sheet that have undergone recrystallization also increases in the range CR-50% to CR-85%. Throughout this procedure, the grains experience changes in deformation, some partial recrystallization, and ultimately full recrystallization [14]. At this stage, the grains are relatively small and uniform in size.

If the annealing temperature is increased to 500 °C, full recrystallization takes place regardless of whether the deformation is 50%, 70%, or 85%. However, after complete recrystallization, some grains grow abnormally and become relatively coarse, and their distribution is not even. The coarse grain size exceeds 200 μm.

**Figure 4:**
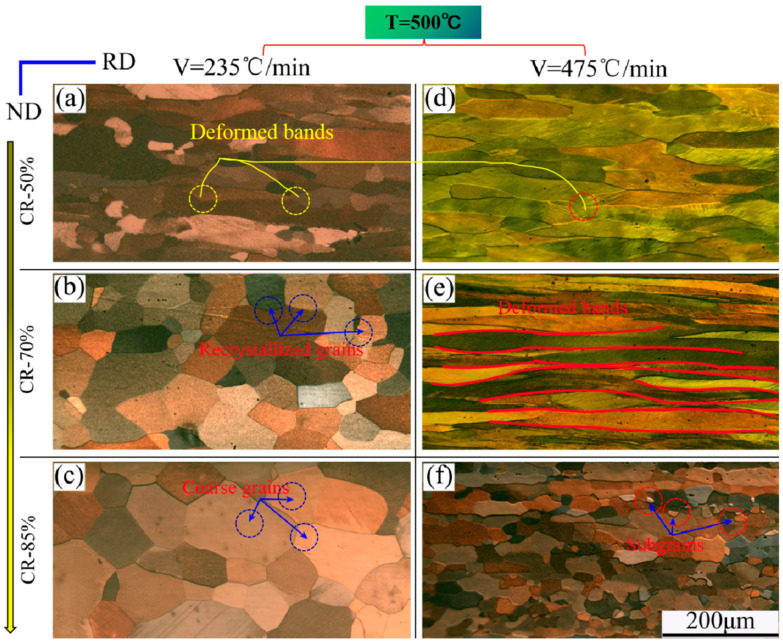
POM image of microstructure under annealing temperature of 500 °C, (**a**–**c**) V = 235 °C/min and CR-50%, CR-70%, CR-85%, respectively; (**d**–**f**) V = 475 °C/min and CR-50%, CR-70%, CR-85%, respectively.

In order to further investigate the impact of heating rate on microstructure, the rate of heating was increased, and annealing was conducted at rates of 235 °C/min and 475 °C/min. The annealing process aimed to reach a target temperature of 500 °C. It was observed that for the same degree of deformation, a higher annealing heating rate resulted in a lower recrystallization fraction. Based on previous research and related mechanisms [25], it can be concluded that a higher heating rate inhibits recrystallization, as the plate does not have sufficient time to recrystallize, resulting in most grains remaining in the deformation state, as is shown in Figure 4. However, the recrystallization process is enhanced with an increase in cold-rolled deformation, resulting in a higher fraction of recrystallization. This effect is particularly noticeable at CR-85%, where almost complete recrystallization takes place in the grain space.

**Figure 5:**
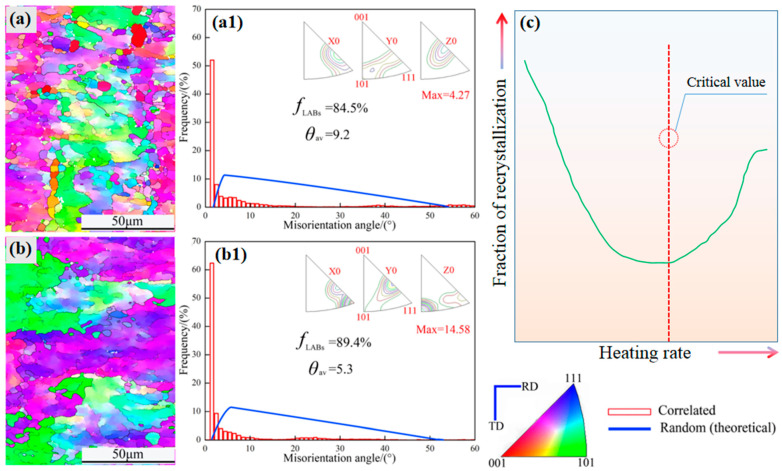
(**a**,**b**) EBSD map of annealed samples (190 °C with 20 °C/min and 30 °C/min, respectively). (**a1**,**b1**) Misorientation angle and (**c**) fraction of recrystallization vs. heating rate.

To examine the microstructure and grain boundary characteristics in a more detailed way, we investigated the morphology of image orientation using an FEI Nova 400 field emission microscope to observe and analyze aluminum plates with different deformation values. It can be seen that no obvious recrystallization occurs when the annealing temperature is 190 °C. As can be seen from Figure 5, as the heating rate increases, the degree of recrystallization becomes smaller, and it is even difficult to recrystallize. When the heating rate is 20 °C/min, most areas of the aluminum foil are still in a recovery state, showing deformed microstructure. Only a few preferential nucleated grains are recrystallized, and the recrystallized grain size is very small (shown in Figure 5a). The proportion of low-angle grain misorientation angle is 84.5%, and the average misorientation angle is 9.2°, which is consistent with the fact that most areas of the aluminum foil have deformed microstructure (Figure 5(a1,b1)). When the heating rate is increased to 30 °C/min, due to the further shortening of the annealing time, it is difficult for the aluminum foil to recrystallize, and the aluminum foil is essentially in the deformed structure. At this time, the proportion of low-angle misorientation angle is 89.4%. The average misorientation angle is 5.3°, so it can be considered that the aluminum foil grain boundary structure is dominated by low-angle grain boundaries (Figure 5(a1,b1)).

The results of Figure 5, which are in complete agreement with those depicted in Figure 4, indicate a congruent recrystallization behavior. Additionally, the grain boundary characteristics are distinctly observable through the progression of dislocation, commencing with the creation of deformation bands and culminating in the disappearance of grain nucleation and growth.

In fact, in the context of annealing, heating rate, and cold rolling deformation, it has been observed that at an annealing temperature of 500 °C, a deformation of CR-70% occurs, which results in complete recrystallization of grains, leading to a more uniform, fine, and equiaxed grain size. However, when the deformation degree is increased to 85%, grain coarsening occurs after the completion of recrystallization. This is primarily due to higher deformation energy storage [6,11], which provides the driving force for the secondary growth of grains after recrystallization, leading to rapid grain growth and coarsening (Figure 4c). Figure 5c indicates that a critical heating rate is the key to the recrystallization behavior. If you want to obtain a uniform and high fraction of cubic texture, then the heating rate should be chosen at the critical point.

### 3.2. Crystal Orientation Analysis

**Figure 6:**
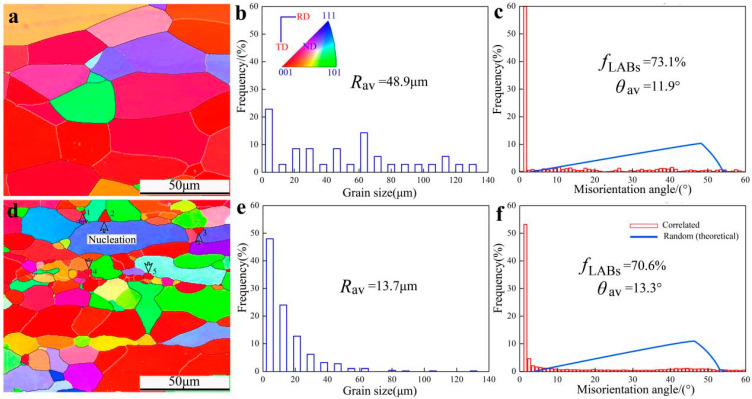
EBSD map, grain size distribution, misorientation angle of the annealing temperature of 500 °C, (**a**–**c**) V = 10 °C/min; (**d**–**f**) V = 25 °C/min.

The investigation of micro-grain orientation imaging has the potential to unveil the progression of microstructure and micro-texture in aluminum foil during annealing, as well as the underlying mechanism of recrystallization. Figure 6 expounds the characteristics of crystal orientation, grain size distribution, and misorientation within microregions of the aluminum sheet.

The ability to selectively nucleate the planar (001) grains has been discovered. The deformation storage energy is released as a result of the removal of dislocation during annealing, allowing the grain size of plane (001) to increase quickly and surpass that of plane (111), as shown in Figure 6a,d, the numbers 1–5 represent the (001) oriented grain, and all are subcrystalline distributed in the orientation space, and the observation surface was RD/TD (RD-Rolling direction, TD-Transverse direction, ND-Normal direction). The previously mentioned information is contrasted with the phenomenon of abnormal grain development during quick annealing. Based on the theory of recrystallization, it can be assumed that the mechanism of recrystallization might range from continuous to discontinuous. Recrystallized grains from fine to excessively coarse individual grains, exhibit irregularities in the final structure as a result of the crystallization transition, such as unequal distribution.

The average grain size is 48.9 μm and the fraction of low-angle boundary is 73.1% when the annealing heating rate is 10 °C/min (Figure 6b,c), while the average grain size is 13.7 μm and the fraction of low-angle boundary is 70.6% when the annealing heating rate is 25 °C/min (Figure 6e,f), indicating that the annealing heating rate has a significant impact on the recrystallization process. The recrystallization grains become finer as the heating rate increases. The grain boundary fraction decreases with decreasing grain size in the same direction.

**Figure 7:**
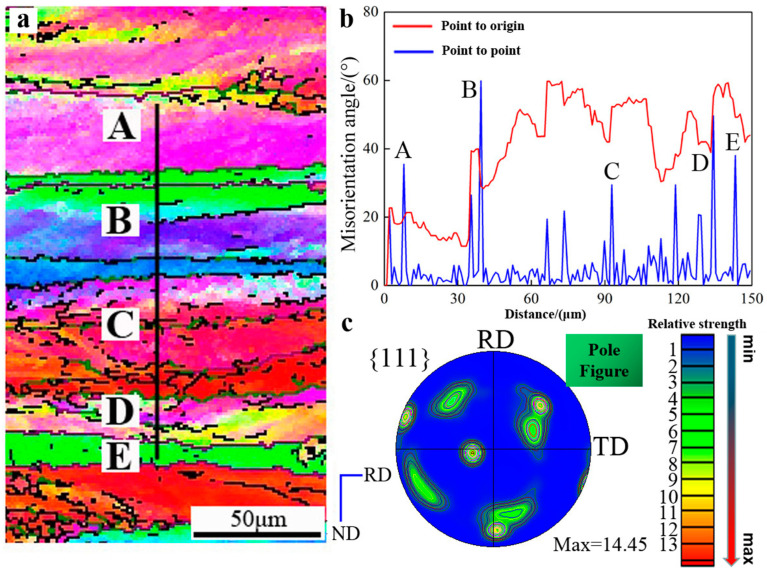
(**a**) EBSD micrograph of the five regions (A–E) separated by HAGBs under CR-70%. (**b**) Relative and accumulative misorientation profiles along the vertical line in (**a**) showing the cross-over of the A–E regions (taken parallel to ND). (**c**) {111} pole figure of area in (**a**) showing random texture components and relative strength.

Figure 7 depicts how grain boundary features affect the cold-rolled aluminum’s deformation behavior at the grain boundaries. High-angle grain boundaries (HAGBs) were used to distinguish the five areas labelled A through E. In Figure 7b, the relative misorientation along the vertical line in Figure 7a is displayed, with just six HAGBs visible over a 150 μm distance. While the boundaries between the regions B/C and C/D are low-angle, those between the regions A/B and D/E are high-angle. As seen in Figure 7b, the volume fraction of LAGBs (less than 15°) is greater than that of HAGBs (greater than 15°). From one point to the next along the black line, the misorientation is obtained.

The cumulative misorientation profile in Figure 7a demonstrates the significant orientation changes in this area of the microstructure as well as the presence of HAGBs along the line. They lack thin transition bands (~25 μm) and are typical deformation banding structures. The {111} pole figure of the cold-rolled sheet demonstrates that the S, Goss, and Brass textures were the predominant ones, as shown in Figure 7c.

### 3.3. Hardness Testing

**Figure 8:**
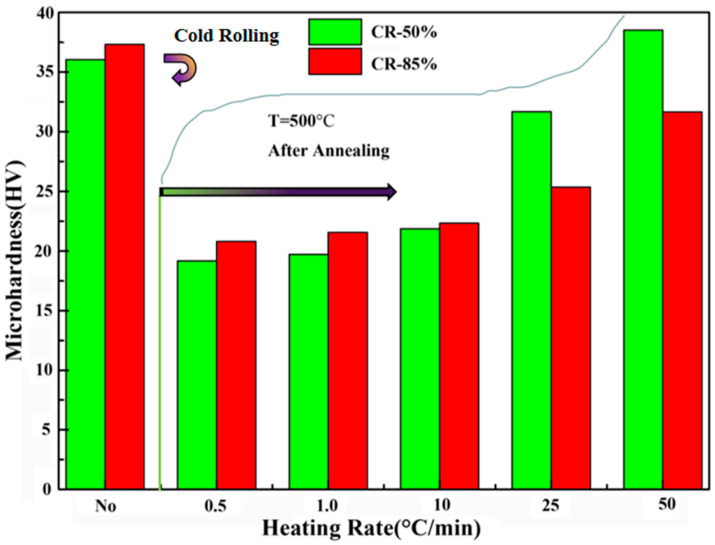
The relationship between microhardness and heating rate under the different cold rolling deformations.

In order to establish the relationship between microstructure and performance, we carried out a hardness test. The present study conducted a microhardness test on aluminum plates that underwent cold-rolling and annealing processes. The findings indicate that the hardness of cold-rolled aluminum plates is elevated due to the work hardening effect. However, after annealing, the hardness of aluminum plates decreases due to the softening effect of the process [19]. Moreover, the reduction in hardness is more pronounced with a decrease in heating rate, as shown in Figure 8.

In the state of cold rolling, the CR-85% aluminum sheet exhibits a higher level of hardness compared to CR-50%, with a value of 38HV. Even when subjected to a heating rate of 50 °C/min, the grain structure remains in the deformed state, which is nearly consistent with the hardness observed in the cold-rolled state. This highlights the significance of the heating rate as a crucial factor influencing the hardness value. Clearly, the heating rate has a certain relationship with the microstructure evolution and property changes, and will also affect the material properties. In particular, this has serious effects on the capacitor aluminum foil via control of the heating rate.

The microstructure morphology and recrystallization behavior are significantly influenced by various factors such as the degree of cold rolling deformation, annealing temperature, and annealing heating rate. These factors play a crucial role in determining the difference between the initial recrystallization driving force and the secondary grain growth driving force [17,20]. The latter is primarily attributed to the consumption and retention of deformation stored energy.

## 4. Discussion

By studying the structure and performance changes of capacitor aluminum foil, the influencing factors of its electric storage performance are discussed. The purpose of this is to investigate how to increase its electric storage capacity and facilitate better design for a capacitor in the microelectronics industry.

The effectiveness of aluminum electrolytic capacitors is evaluated based on the size of their electric capacities [18,22], which is mainly determined by the specific capacitance per unit area of the anode aluminum foil. The formula for this capacitance described below plays a significant role in determining the value of the metric.

$$C=8.855\times 10^{-12}\times \frac{\varepsilon S}{t}$$

**Figure 9:**
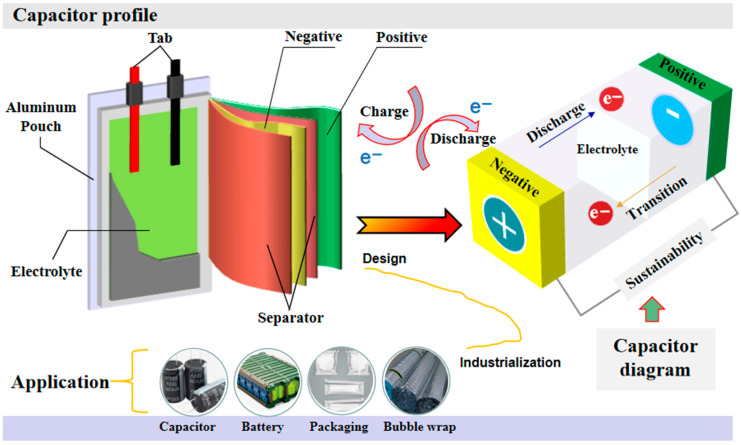
The diagram of structure, charging and discharging process for electrolytic capacitor.

The capacitance of anode aluminum foil is determined by the dielectric constant, electrode surface area, and thickness of the Al_2_O_3_ insulation layer. The capacitance is directly proportional to the dielectric constant and surface area, but inversely proportional to the thickness of the insulation layer. The dielectric constant typically ranges from 7 to 10, while the thickness ranges from 1.0 to 1.5 nm/V. To increase the capacitance of aluminum electrolytic capacitors, it is important to increase the effective surface area of the high-voltage anode aluminum foil. This is crucial for achieving miniaturization and high capacitance, as shown in Figure 9.

Currently, aluminum foil is widely utilized in electric products, which is due to its excellent thermal and electrical conductivity. It is primarily employed in various applications such as capacitor anodes, power batteries, food packaging shells, and heat insulation air cushion films [26,27].

To enhance the surface area of aluminum foil in manufacturing capacitors, etching technology is utilized to create surface irregularities without increasing its volume. This leads to an improved effective surface area. The corrosion process of the aluminum foil occurs uniformly and in a structured manner in the crystallographic direction, particularly in the (100) <001> direction, which provides the best corrosion channel. This results in a greater increase in effective surface area. Therefore, to increase the effective surface area S of high-voltage anode aluminum foil, it is necessary to increase the cubic texture or (100) surface fraction.

The research presented in this paper has shown that a slow heating process can increase the effective surface area of aluminum foil by promoting a higher cubic texture content and uniform spatial distribution [13,23]. It has also been found that the grain size of the cube texture should be moderate and uniform to achieve the desired outcome. Zhang et al. [16] discovered through experimentation that the optimal grain size range for aluminum foil is between 60 and 200 μm. If the grain size is too small, it can cause inter-crystalline corrosion and surface peeling, leading to a decrease in aluminum foil thickness and surface area, which ultimately reduces the specific capacitance. Conversely, if the grain size is too large, it can decrease the fraction of cubic texture and specific capacitance.

## 5. Conclusions

A novel experiment process of controlling heating rate to produce an aluminum plate through cold rolling and annealing processes was investigated. The study focused on analyzing the microstructure morphology, recrystallization behavior, grain size, and other related factors. Based on the results, certain conclusions were made, as listed below:

(1) The cold rolling reduction rate, annealing temperature, and heating rate have a common impact on the microstructure, recrystallization behavior, and secondary grain growth during the annealing process.
(2) The recrystallization temperature increases with an increase in the heating rate, while the recrystallization fraction decreases. Additionally, a decrease in the recrystallization temperature is observed, which means that the heating rate is a very important factor affecting the recrystallization.
(3) In order to ensure the uniformity of microstructure and obtain a high cubic texture content, it is necessary to determine a reasonable cold rolling reduction rate, appropriate annealing temperature, and appropriate heating rate for this study. It is a very important reference to guide the actual production of enterprises in the future.

## Data Availability

Not applicable.
